# The Treatment of Aneurism

**Published:** 1899-01-21

**Authors:** 


### THE TREATMENT OF ANEURISM.

IT is interesting to note how many surgical processes which have been rejected after careful trial years ago are now giving good promise when tried afresh under modern conditions of asepsis. Much as the old waste rubbish heaps of the earlier gold diggers often repay working over again by aid of modern machinery, so do some surgical experiments of the past yield good results when tried afresh in the light of the newer knowledge as to antisepsis and technique. A good example of this is to be found in the revival of the plan of treating aneurism by the introduction of fine wire into the sac, and the passage of the galvanic current through the wire so employed. There is but little doubt that some of the non-success which attended the introduction of wire in former times was due to sepsis, and that the success of some recently reported cases has hinged on the fact that it is now possible to make use of the coagulum-forming influence of the wire, of the galvanism, and of the irritation of the lining of the sac, without risking those septic complications or that ulceration of the wall which were so much to be dreaded in former times.

